# Clinical Efficacy of a Novel Topical Formulation on Periorbital Dark Circles: An Objective Analysis

**DOI:** 10.1111/jocd.70326

**Published:** 2025-07-08

**Authors:** Robert Thomas Brady, Sabrina Shah‐Desai

**Affiliations:** ^1^ Department of Ophthalmology Addenbrooke's Hospital Cambridge UK; ^2^ Perfect Eyes Ltd London UK

**Keywords:** dark circles, hyperpigmentation, periorbital

## Abstract

**Background:**

Hyperpigmentation and periorbital dark circles remain challenging dermatological concerns due to their multifactorial etiology, including vascular, pigmentary, and structural components. This study evaluates the efficacy of a novel multi‐action topical formulation designed to target hyperpigmentation and skin aging through a synergistic blend of active ingredients.

**Objective:**

To assess the objective and clinical effects of a dermatological composition containing niacinamide, arbutin, tranexamic acid, ubiquinone, a DHEA‐like component, curcuma oily extract, and marine exopolysaccharides on periorbital hyperpigmentation and skin quality.

**Methods:**

Subjects with visible under‐eye dark circles applied the formulation twice daily over a treatment period of 6 weeks. The evaluation included instrumental quantitative assessments of skin pigmentation along with standardized imaging. A separate safety study and cosmetic efficacy questionnaire of patient experience were completed.

**Results:**

The formulation demonstrated statistically significant improvements in pigmentation intensity. The twice daily use of the under‐eye serum resulted in an average overall reduction in under‐eye hyperpigmentation of 47.94%, with no adverse effects observed.

**Conclusion:**

This novel topical composition offers a safe and effective multi‐targeted approach for improving periorbital hyperpigmentation and promoting overall skin rejuvenation. The synergistic action of its active components supports its use in clinical and aesthetic dermatology for the treatment of dark circles and age‐related changes.

## 1 Introduction

Dark circles (periorbital hyperchromia) of the lower eyelid are an increasingly prevalent cosmetic concern in patients presenting to the cosmetic dermatology clinic. Evidence suggests that hyperpigmentation of the eyelids is caused by aging changes, melanisation of the dermis and/or congested blood flow in the periorbital area [1, 2].

Delicate undereye skin is susceptible to environmental stresses that increase oxidative stress by free radicals such as UV exposure, smoking, and air pollution that result in damage to cellular and extracellular components. Moreover, environmental stresses may deplete the moisture of the skin, exacerbating the appearance of fine lines and wrinkles, combined with decreasing collagen and hyaluronic acid concentrations in the skin over the years. It is easy to see why older patients may suffer from dark circles.

Free radicals that can damage DNA, alter the cytoskeleton, and trigger the release of inflammatory mediators can be neutralized in the body by antioxidants. Natural antioxidants in the body include enzymatic antioxidants such as superoxide dismutase, catalase, and glutathione peroxidase; while known non‐enzymatic antioxidants that may assist the skin in scavenging UV‐induced free radicals include vitamin A, vitamin C, and vitamin E [3]. Melanin, produced by melanocytes upon exposure to UV rays, can act as an antioxidant but also causes the skin to appear darker. In some cases, this darkening is uneven and results in irregular hyperpigmentation. This is particularly true in older patients whereby melanocyte distribution becomes less regulated by the body. Hyperpigmentation is also a feature of systemic illnesses including Addison's, Cushing's, and coeliac diseases. It can also result secondary to infections (tinea) or exposure to chemicals such as salicylic acid, bleomycin, and cisplatin.

Hyperpigmentation treatments center around topical agents to support hypopigmentation of the problem area and adjuncts to improve localized blood flow. These treatments primarily target tyrosinase but fail to offset the offending triggers or minimize other signs of aging by reducing the appearance of fine lines and wrinkles. The purpose of this study was to investigate the effectiveness of a novel blend of topical actives on dark circles of the lower eyelid using quantitative image analysis.

A multi‐action dermatological composition has been developed that acts as an anti‐aging brightening complex that imparts a lightening, antioxidizing, and lifting effect when applied topically to the skin. The complex comprises niacinamide, arbutin, tranexamic acid, ubiquinone, ascorbyl glucoside, a DHEA‐like component, curcuma oily extract, and marine exopolysaccharides.

The purpose of this study was to investigate the effectiveness of a novel blend of topical actives on dark circles of the lower eyelid using quantitative image analysis. We found that blends of topical actives significantly improve the appearance of dark circles within 6 weeks of use.

## 2 Methods

### 2.1 Study Design

This was an open‐label, single‐arm, prospective study carried out in accordance with the principles of the declaration of Helsinki, International Conference on Harmonization Good clinical practice (ICH‐GCP). Recruited patients had baseline facial imaging completed and repeat imaging at a 6‐week endpoint. Within this time period, patients would apply the novel eye serum to their under‐eye areas. All subjects were supplied with a topical eye serum containing a number of active ingredients that include: Niacinamide, natural arbutin, curcuma oily extract, DHEA derivatives, Co‐enzyme Q10, and tranexaminc acid (Table 1). Subjects were instructed to apply the serum to their under‐eye areas twice a day for a period of 6 weeks.

**TABLE 1:** Composition of novel eye‐serum complex.

Ingredient	Description
Niacinamide (vitamin B3)	A powerful antioxidant that enhances the prevention of pigmentation, improves the lightening effect on brown spots, and works in synergy with tranexamic acid for visible improvements of dark circles
Natural arbutin	Strongly inhibits tyrosinase activity, which is the key enzyme of the melanin pigment synthesis process
Curcuma oily extract	A powerful anti‐inflammatory and antioxidant which targets blemishes to fade away naturally and give the skin a more even‐toned appearance
DHEA derivative	Increases the density and elasticity of the skin to counteract the papery appearance of aging skin and epidermal atrophy, as well as improving skin brightness
Co‐enzyme Q10	A family of lipid soluble antioxidants that work together to protect cell membranes from free radical damage and collagen breakdown. Reduces dark spots and evens out skin tone by blocking tyrosinase, limiting the synthesis of melanin
Tranexamic acid	Inhibits UV‐induced melanin synthesis by blocking the interaction between keratinocytes and melanocytes and ensures that long term accumulative results are not compromised by UV exposure

They were allowed to engage with their usual face cleansing products but were not to apply any products to their under‐eye area. At the endpoint, a comparative quantitative analysis between baseline and endpoint metrics was completed. A second, separate clinical trial investigating product safety for our serum was completed by Ocealys Laboratoire, Plouzané, France, on 20 female volunteers using a twice daily application for 6 weeks as described above. All 20 subjects completed the 6‐week study and were assessed by a dermatologist and an ophthalmologist. These patients also completed a cosmetic efficacy questionnaire surrounding their subjective experience of the serum.

### 2.2 Patient Selection

Recruited patients were adults with concerns regarding the appearance of under‐eye dark circles. Subjects who had not participated in similar investigations and who were willing to agree to the study protocol were included. Subjects with active dermatological or ophthalmological disease, including blepharitis, were excluded. Patients identified to be allergic to any formulation ingredients, pregnant, or breast feeding were also excluded.

In total, 53 subjects with dark circles of the lower eyelids were recruited for the study and informed consent was obtained. Patients had their Fitzpatrick skin types determined and found to have Type 1 9%, Type 2 26%, Type 3 15%, Type 4 13%, Type 5 26%, and Type 6 9%, illustrating a fair distribution of skin types in the study. The second safety study of 20 female volunteers also had a varied range of skin types 1–4.

### 2.3 Image Acquisition

Photographs were taken on the Aram Huvis API 100 camera (Aram Huvis Co. LTD., Gyeonggi‐do, South Korea) of the infraorbital area of both the right and left eyes of each patient at week 0 and week 6. This digital skin analysis camera is equipped with a high‐resolution complementary metal‐oxide‐semiconductor (CMOS) sensor that captures detailed images of the skin for visual assessment rather than direct quantitative color measurement. This is achieved using a specialized light source and CMOS sensor to capture high‐resolution images. An analysis algorithm is used to quantify erythema and skin sensitivity. Quantitative results allow the practitioner to monitor for changes over time and assess the impact of any applied treatments.

Using the Aram Huvis scoring system, the melanin concentration was measured on the darkness and width of melanin in the infraorbital area. The percentage reduction in hyperpigmentation from week 0 to week 6 was then determined. All measurements were taken under controlled conditions, as a fixed point on the lower lid to prevent any variations in the analysis of the images. The values represent the amount of melanin in the infraorbital area, with lower numbers representing lower levels of melanin and pigmentation, whereas higher numbers represent higher levels of melanin and pigmentation.

### 2.4 Statistical Analysis

Statistical analyses were performed using GraphPad Prism (Version 5.0) (GraphPad Software, CA, USA) software. All data are expressed as means ± standard error with overlying data points. One‐way analysis of variance (ANOVA) was used with a Bonferroni test to compare between groups. A probability value of 95% (*p* < 0.05) was used to determine significance.

## 3 Results

### 3.1 No Adverse Events Reported by Participants

Of the 53 participants, no subjects reported any adverse effects such as skin irritation, allergic reaction, or ocular irritation over the 6‐week study period. In the second safety study completed by Ocealys Laboratoire, Plouzané, France, all subjects completed the 6‐week study, and there were also no adverse events reported. The results of the cosmetic efficacy questionnaire confirmed the dermatological composition provides skin brightening, lightening, lifting, and smoothing effects when applied to the eye area of the user (Table 2).

**TABLE 2:** Tabulated results of the safety of use and cosmetic efficacy questionnaire on 20 female volunteers after twice daily use for 6 weeks. Performed by Ocealys Laboratoire, Plouzané, France.

Question	Positive answers (% of subjects)
The texture of the product is pleasant	100
The product penetrates easily	95
The product does not leave the skin oily	100
The product leaves the skin instantly brighter	67
The product revives the radiance of the skin instantly	71
The product helps reduce the appearance of fine lines and wrinkles	62
The product visibly reduced the appearance of eye bags	56
The product leaves the complexion of the skin around the eyes more uniform	76
The product visibly improved the tired appearance of the skin around the eyes	52
The product leaves the skin soft and smooth	95
The product decreases the appearance of pigment and dark spots	62
The product leaves the skin hydrated	90
The product leaves the skin lighter	67
Skin looks revitalized	67
The product leaves the skin brighter	62
The product leaves the skin looking healthier	71
The product provides a lift effect	57

### 3.2 Topical Treatment Resulted in Less Pigmentation After 6 Weeks

In the 53‐subject study, the twice daily use of the under‐eye serum resulted in an average overall reduction in under‐eye hyperpigmentation of 47.94%. Separately, this correlated to a right eye reduction of 48.44% and a left eye reduction of 47.43%, respectively.

**FIGURE 1:**
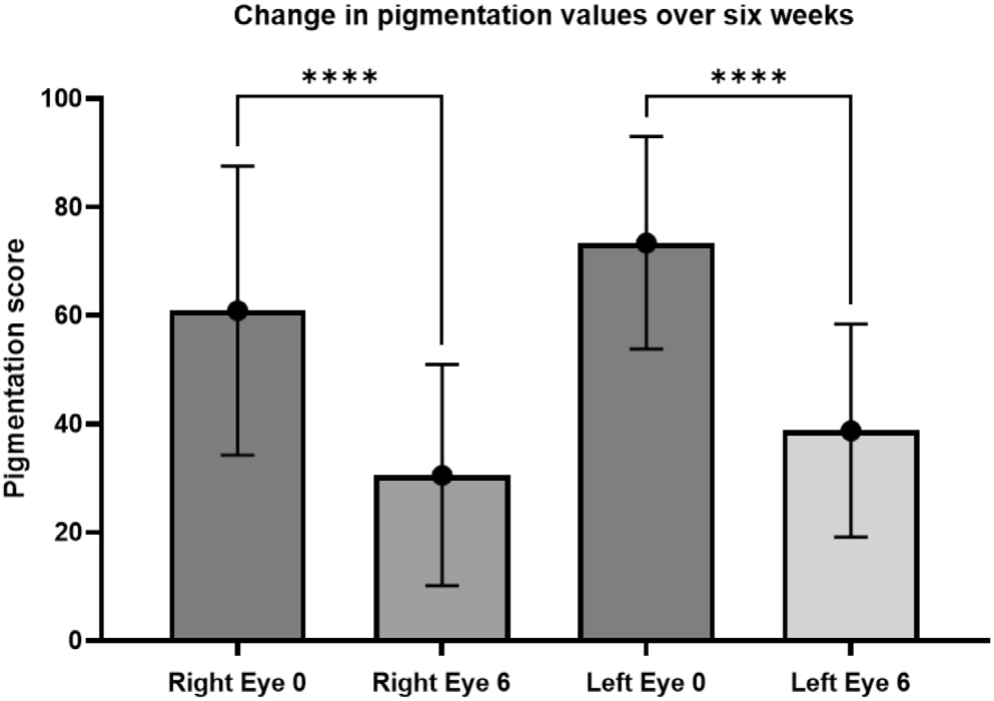
A graphical illustration of changes in pigmentation score from week 0 to week 6 for both the right and left eye. ****p < 0.0001.

The results are shown in Table 3, and the average percentage reduction in hyperpigmentation for each eye is tabulated in Table 4. Analysis determined a statistically significant reduction (*p* < 0.0001) in both right and left eyes at week six when compared to week 0 (Figure 1).

**TABLE 3:** Reduction of pigmentation scores for all patients of both eyes from week 0 baseline and week 6 study end.

Participant	Right eye week 0	Right eye week 6	Right eye percentage reduction (%)	Left eye week 0	Left eye week 6	Left eye percentage reduction (%)
1	34	12	64.71	39	23	41.03
2	20	1	95.00	43	12	72.09
3	88	23	73.86	90	19	78.89
4	80	50	37.50	90	50	44.44
5	1	1	0.00	4	1	75.00
6	81	30	62.96	90	64	28.89
7	41	1	97.56	63	24	28.89
8	20	16	20.00	51	19	62.75
9	53	13	79.93	54	22	59.26
10	31	19	38.71	55	32	41.82
11	90	44	51.11	90	52	42.22
12	29	13	55.17	53	17	67.92
13	36	20	44.44	87	43	50.57
14	42	20	52.38	90	63	30.00
15	75	39	48.00	90	57	36.67
16	68	48	29.41	83	60	27.71
17	45	21	53.33	51	46	9.8
18	70	61	12.86	90	67	25.56
19	90	21	76.67	90	46	48.89
20	35	32	8.57	52	59	−13.46
21	80	58	27.50	90	70	22.22
22	63	37	41.27	90	58	35.56
23	58	29	50.00	90	30	66.67
24	31	17	45.16	90	42	53.33
25	47	38	19.15	81	50	38.27
26	44	41	6.82	74	51	31.08
27	90	34	62.22	90	43	52.22
28	90	14	85.44	90	17	81.11
29	90	67	35.56	90	67	25.56
30	90	74	17.78	78	65	16.67
31	90	75	16.67	90	77	14.44
32	90	78	13.33	73	65	10.96
33	32	10	68.75	64	10	84.38
34	44	41	6.82	46	41	10.87
35	90	41	54.44	65	41	36.92
36	90	32	64.44	90	32	64.44
37	90	50	44.44	84	50	40.48
38	81	50	38.27	77	50	35.06
39	35	10	71.43	35	18	48.57
40	90	64	28.89	90	64	28.89
41	81	37	54.32	81	37	54.32
42	59	13	77.97	59	13	77.97
43	69	9	86.96	72	32	55.56
44	80	13	83.75	80	13	83.75
45	80	14	82.50	80	20	75.00
46	90	23	74.44	90	23	74.44
47	90	49	45.56	90	49	45.56
48	11	2	81.82	83	11	86.75
49	47	15	68.09	52	45	13.46
50	31	29	6.45	90	30	66.67
51	84	40	52.38	70	32	54.29
52	20	24	−20.00	48	25	47.92
53	40	5	87.50	62	7	88.71

**TABLE 4:** The average percentage reduction in hyperpigmentation for each eye.

Average right eye percentage reduction (%)	48.44
Average left eye percentage reduction (%)	47.43
Average overall reduction (%)	47.94

**FIGURE 2:**
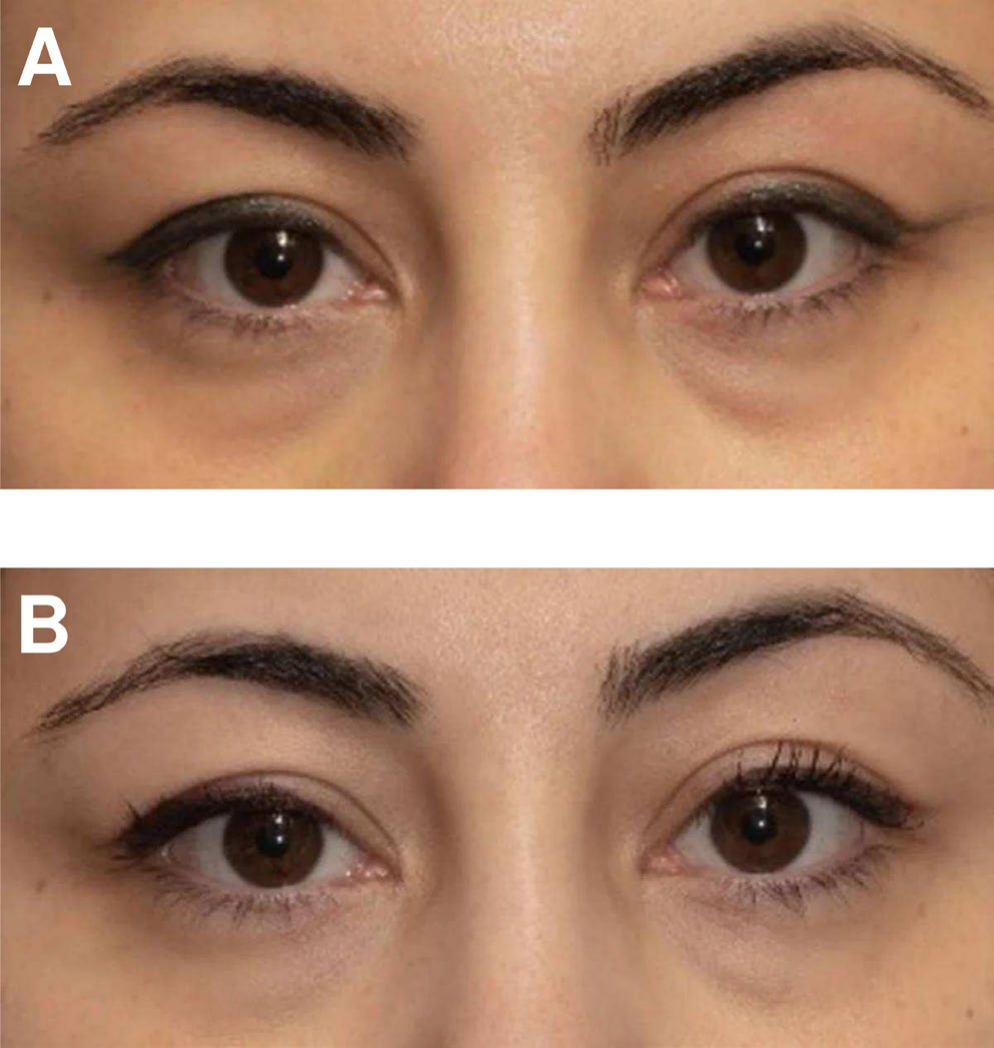
Representative photographs of a patient at week 0 (A) and taken at week 6 (B).

**FIGURE 3:**
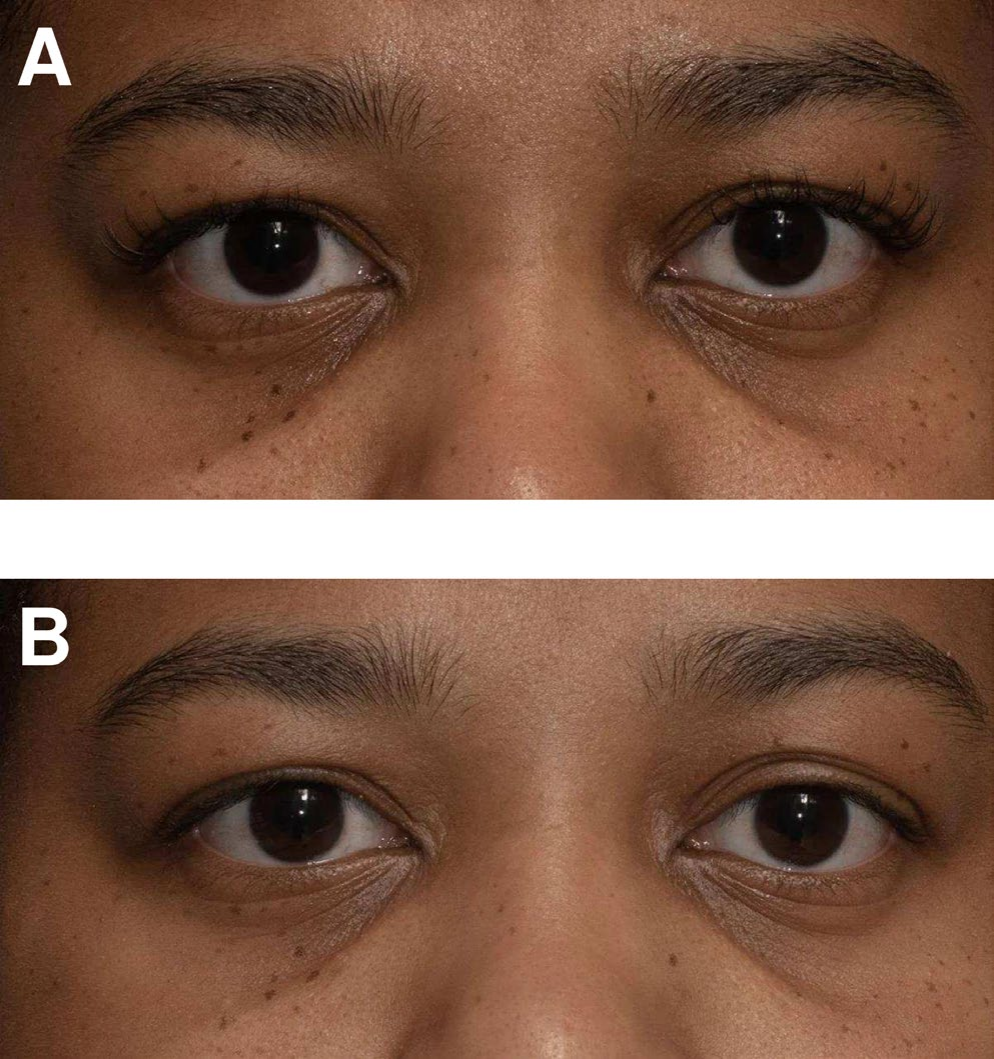
Representative photographs of two patients at week 0 (A) and taken at week 6 (B).

The results demonstrate that an eye serum containing a blend of naturally derived and synthetic extracts of the novel blend achieved global skin rejuvenation by significantly improving the appearance of periorbital hyperpigmentation. Representative images of two patients are included at week 0 (before) and week 6 (after) (Figures 2 and 3).

### 3.3 Aram Huvis Analysis Illustrating Changes Observed in Patient's Skin Upon Topical Treatment After 6 Weeks

Using the inbuilt analysis algorithm, it was possible evaluate patient skin metrics including sensitivity, pore appearance, wrinkles, acne, and melanin. These metrics are graded on a three‐point scales: Good, Care needed, Intensive care. Included are the representative outputs for two sample patients of the cohort. Both are females in their 30's.

**FIGURE 4:**
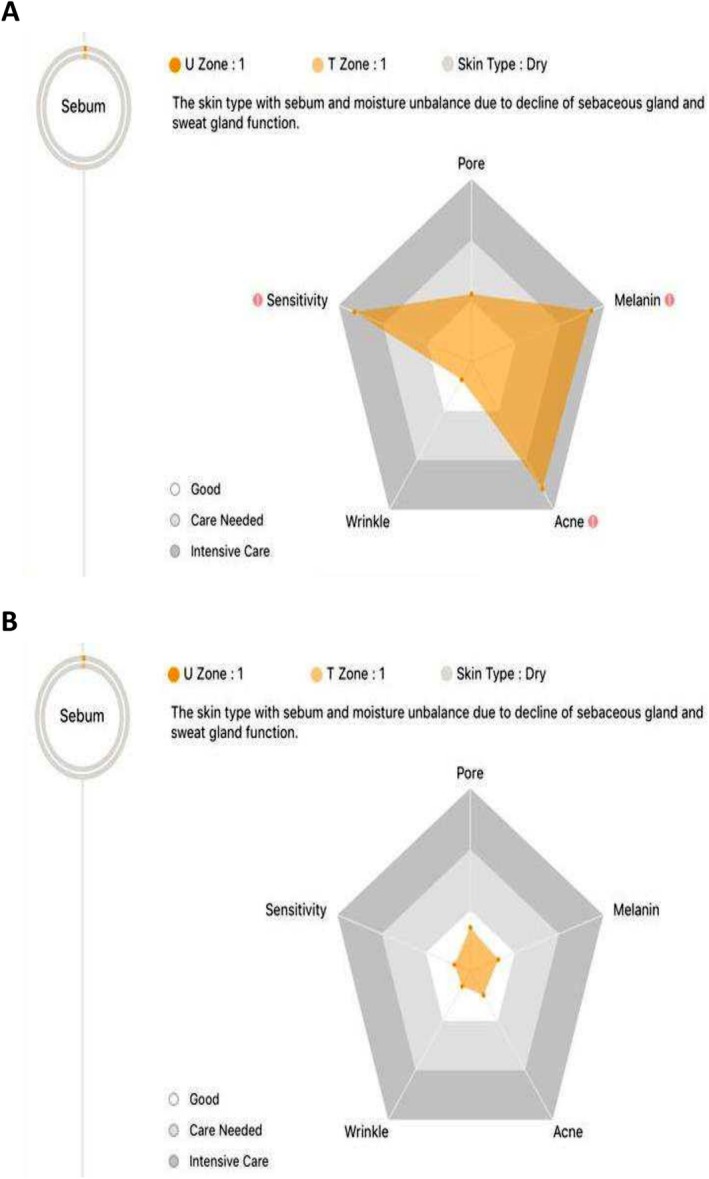
Aram Huvis analysis showed improvements in patient metrics after topical treatment for 6 weeks for first representative patient. (A) Three metrics graded as ‘intensive care’ at week 0. (B) All metrics graded as ‘good’ at week 6.

**FIGURE 5:**
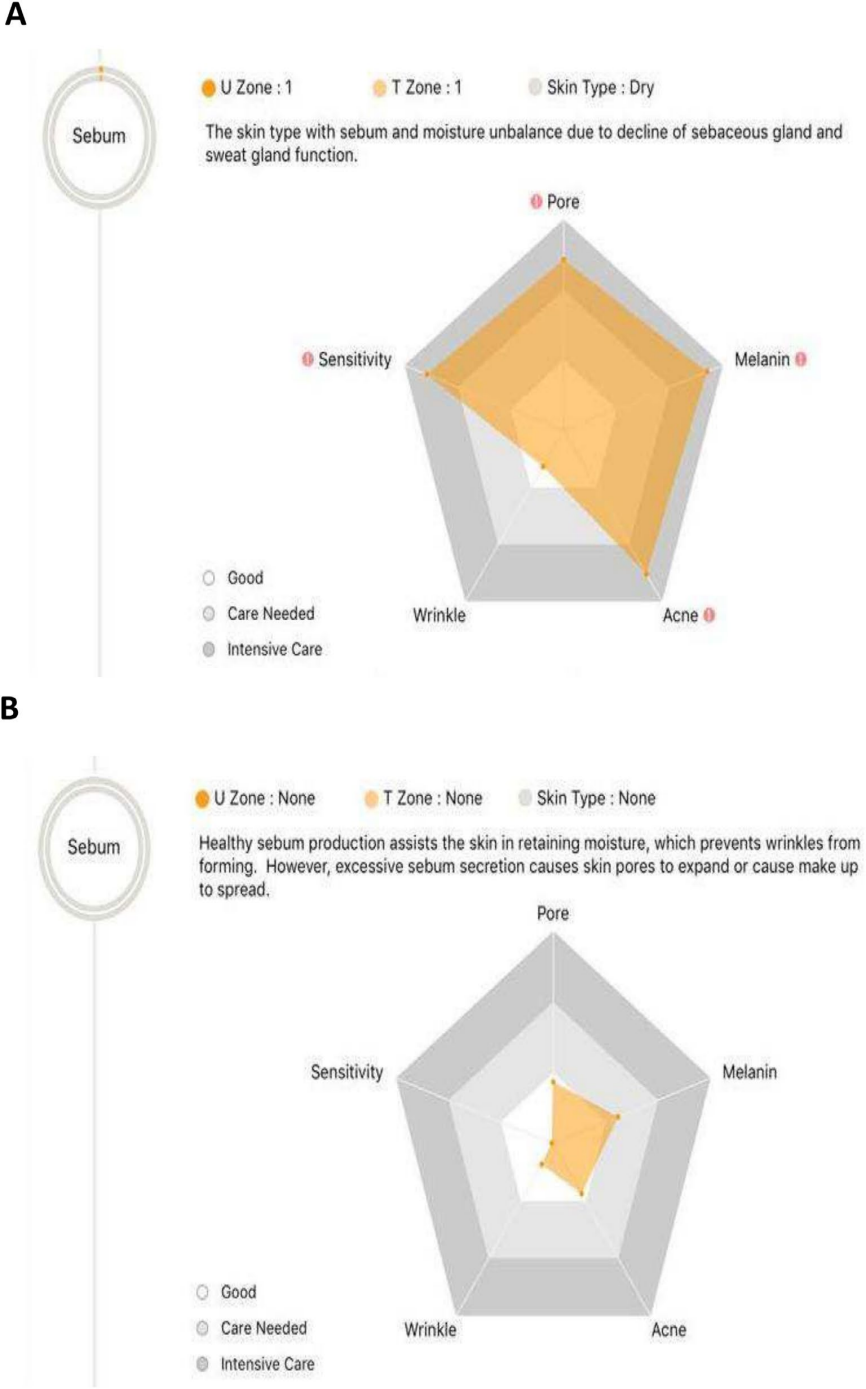
Aram Huvis analysis showed improvements in patient metrics after topical treatment for 6 weeks for second representative patient. (A) Four metrics graded as ‘intensive care’ at week 0. (B) Reduction in grading for all metrics. Most by two grades to reach ‘good’ by week 6.

The first patient was graded ‘intensive care’ on four of the five metrics. After 6 weeks topical treatment with this eye serum, all metrics showed improved with three of the four outputs now graded as ‘good’ (Figure 4). The second patient at week 0 had three areas graded as ‘intensive care’ and one as ‘care needed’. At week 6 she was found to have a grading of ‘good’ in all metrics (Figure 5).

## 4 Discussion

This study introduces a novel serum that can significantly reduce the appearance of periocular pigmentation. Commonly referred to as ‘dark circles’, these represent a significant aesthetic concern for many individuals. Whilst the etiology is incompletely understood, a multi‐factor cause is postulated that encompasses intrinsic and external factors. Commonly cited causes include excessive pigmentation, vascular perfusion and lifestyle factors including stress and sleep deprivation [4, 5].

The treatment options for dark circles are diverse, reflecting the multifactorial nature of the condition [6]. Non‐surgical options are frequently employed, and many aim to reduce melanin pigment production and stimulate blood flow around the periorbital area [7]. Treatments include topical agents, injectable fillers, and various laser therapies. Dermal fillers can restore volume to thin skin, whilst lasers target pigmentation and vascularity [8]. Newer modalities include the injection of platelet rich plasma (PRP) to rejuvenate a patient's skin with their serum factors [9, 10]. Surgical interventions, such as lower blepharoplasty, are considered for more severe cases where structural issues contribute significantly to the appearance of dark circles. This procedure can reposition or remove excess fat and skin, addressing both shadowing and volume loss. Limitations of these modalities include both cost and what is acceptable to the patient. There is therefore a clinical need to develop an acceptable, efficacious, and cost‐effective method of addressing dark circles.

Excessive pigmentation of the eyelids may be attributed to melanin and hemoglobin, which are the primary chromophores in the skin, and their presence can result in changes in skin color. Melanin is produced by melanocytes, and the differences in pigmentation in skin are due to the ratio of eumelanin (brown‐black) to pheomelanin (yellow‐red), as well as the number of melanosomes within the melanocytes. Studies carried out on Fitzpatrick skin types I–III have shown that those with a higher skin type have an increased eumelanin content, compared to those of a lower skin type [11]. Hemoglobin is the dominant protein constituent of red blood cells, which has a primary role of transporting oxygen throughout the body. Oxygenated hemoglobin produces a pink tint in lightly pigmented skin, whereas deoxygenated hemoglobin produces a blue tint in lightly pigmented skin. Thin periocular skin, combined with underlying vascularity, results in shadowing and exacerbates the appearance of dark circles. Moreover, evidence suggests that dark circles appear in areas of the skin where there is congestion or stasis of blood flow [12]. Topical treatments that can modulate the production of melanin and vascularity are therefore a source of interest in managing those patients where hyperpigmentation and vascular shadowing are the main sources of their dark circles.

Topical agents traditionally employed to reduce dark circles include bleaching agents such as hydroquinone and chemical peels using trichloroacetic acid (TCA) and glycolic acid. These agents can target melanin accumulation, but their effectiveness can be inconsistent and are often accompanied by adverse effects, limiting their desirability as treatment options. In contrast, less invasive treatments, like hyaluronic acid fillers, can improve the aesthetic appearance by restoring volume lost due to thinning skin and dehydration. Their use is limited by their temporary duration, requiring minimally invasive injections and associated risks, including loss of vision. There is a need, therefore, for safe and effective topical agents to reduce the appearance of dark circles. Research into topical treatments that include ingredients such as vitamin C, peptides, and botanical extracts has shown potential in enhancing skin quality and reducing dark circles [13]. These data add to the growing body of literature that supports the use of topical agents in managing the appearance of dark circles. Importantly, the serum described within achieves a significant reduction in objective pigmentation whilst also achieving patient satisfaction with only 6 weeks of application.

A number of effective agents have been investigated to date. Ertam et al. in 2008 found that treatment with a 1% arbutin gel significantly reduced pigmentation by 43.5% compared with 7.1% in the placebo group [14]. Hakozaki et al. 2002 determined that a 5% niacinamide cream, when applied for 4 weeks, significantly reduced the pigmented spots by 11% [15]. Studies have further identified novel formulations that incorporate botanical extracts and other bioactive compounds that are aimed at enhancing under‐eye skin health, and combination products containing a blend of actives are reported to be better than single active‐containing products [16]. Both of these agents are included in our eye serum formulation and act synergistically to both reduce melanin production and transport.

There have been numerous formulations of active agents developed with contrasting results. A recent study by Rajabi‐Estarabadi et al., 2023 investigated the application of a topical formulation with tetrahexyldecyl (THD) ascorbate (vitamin C), caffeine, bioactive peptides, and botanical extracts. They reported a reduction in the appearance of dark circles of 12.5% at 4 weeks and 20% after 12 weeks of treatment [17]. Similar results were achieved by Ahmadraji and Shatalebi, 2015, who achieved a 16% response rate in the reduction of dark circles using a counter pad containing caffeine and vitamin K in an emulsified Emu oil base after 28 days [18]. Another study utilizing a serum containing tyrostat, aldavine, and lanachrys 2B achieved a mean reduction in colorimetric melanin values of 12.29% after 3 months use [19]. In comparison, our serum used twice daily resulted in an average objective reduction in under‐eye hyperpigmentation of 47.94%. Our serum builds upon the positive findings of topical arbutin and niacinamide, combined with other active ingredients with varying mechanisms of action to achieve this result. Importantly, the second separate clinical trial investigating product safety for our serum, completed by Ocealys Laboratoire, Plouzané, France, on 20 female volunteers, reported no adverse effects after examination by a dermatologist and ophthalmologist. The results of the cosmetic efficacy questionnaire confirmed the dermatological composition provides skin brightening, lightening, lifting, and smoothing effects when applied to the eye area of the user.

For patients with particularly dark circles, or with multifactorial etiologies, a combination of therapeutic strategies is often recommended. A systematic review has confirmed the effectiveness of combining topical agents with procedural modalities, such as laser treatments and fillers. This holistic approach ensures that both pigmentary and structural concerns are addressed [20].

A limitation of this study is the limited timeframe of 6 weeks. Whilst achieving the study objective within this timeframe, it would be interesting to complete longer term follow‐up as to the duration of effect. It would also be of interest in combining this treatment with surgical approaches to assess for any synergistic effect upon the appearance of dark circles. Another limitation of this study was the unblinded nature to both parties. The introduction of bias was reduced in this study; however, by relying on objective measurements rather than subjective response scales. A study using a placebo, comparator, or internal control where the subject would apply the serum to one side was considered. It was thought that there may be challenges in patient compliance with this approach, as the patient, with aesthetic concerns, may wish to apply the serum to the control side to avoid asymmetry as the serum takes effect, resulting in reduced pigmentation on the treatment side arm of the study.

## 5 Conclusion

This study introduces a novel under‐eye serum for the treatment of dark circles. The most important finding was a statistically significant reduction in pigmentation as demonstrated by objective analysis. Importantly, no adverse events were recorded, and all patients reported positive experiences with use of the serum.

## Author Contributions

Robert Thomas Brady – manuscript preparation and review. Sabrina Shah‐Desai – study conception, data collection, manuscript preparation, and review.

## Ethics Statement

The study was conducted with full adherence to scientific and ethical standards. The design, data collection, analysis, and interpretation were carried out independently by the author without external influence. The study received no third‐party funding. The author affirms that the data presented are a truthful and objective representation of the findings.

## Conflicts of Interest

Dr. Sabrina Shah‐Desai is the founder and director of Dr. Sabrina Limited, which manufactures and sells the eye serum evaluated in this study. This commercial affiliation constitutes a potential conflicts of interest. The study was conducted with full adherence to scientific and ethical standards. The design, data collection, analysis, and interpretation were carried out independently by the author without external influence. The study received no third‐party funding. The author affirms that the data presented are a truthful and objective representation of the findings.

## Data Availability

The data that support the findings of this study are available on request from the corresponding author. The data are not publicly available due to privacy or ethical restrictions.
